# Comparison of *Ganoderma boninense* Isolate’s Aggressiveness Using Infected Oil Palm Seedlings

**DOI:** 10.1007/s12275-023-00040-w

**Published:** 2023-04-25

**Authors:** Mei Lieng Lo, Tu Anh Vu Thanh, Frazer Midot, Sharon Yu Ling Lau, Wei Chee Wong, Hun Jiat Tung, Mui Sie Jee, Mei-Yee Chin, Lulie Melling

**Affiliations:** 1 Sarawak Tropical Peat Research Institute, 94300 Kota Samarahan, Sarawak Malaysia; 2 Faculty of Resource Science and Technology, Universiti Malaysia Sarawak, 94300 Kota Samarahan, Sarawak Malaysia; 3 Advanced Agriecological Research Sdn. Bhd., Kota Damansara, 47810 Petaling Jaya, Selangor Malaysia

**Keywords:** Basal stem rot, Artificial inoculation, Vegetative growth measurement, Disease severity index, Rubber wood block

## Abstract

Basal stem rot incidence caused by a white-rot fungus, *Ganoderma boninense*, is the major disease of oil palm in Southeast Asia. The rate of disease transmission and host damage are affected by variations in pathogen aggressiveness. Several other studies have used the disease severity index (DSI) to determine *G. boninense* aggressiveness levels while verifying disease using a culture-based method, which might not provide accurate results or be feasible in all cases. To differentiate *G. boninense* aggressiveness, we employed the DSI and vegetative growth measurement of infected oil palm seedlings. Disease confirmation was performed through scanning electron microscopy and molecular identification of fungal DNA from both infected tissue and fungi isolated from *Ganoderma* selective medium. Two-month-old oil palm seedlings were artificially inoculated with *G. boninense* isolates (2, 4A, 5A, 5B, and 7A) sampled from Miri (Lambir) and Mukah (Sungai Meris and Sungai Liuk), Sarawak. The isolates were categorized into three groups: highly aggressive (4A and 5B), moderately aggressive (5A and 7A), and less aggressive (2). Isolate 5B was identified as the most aggressive, and it was the only one to result in seedling mortality. Out of the five vegetative growth parameters measured, only the bole size between treatments was not affected. The integration of both conventional and molecular approaches in disease confirmation allows for precise detection.

**Supplementary Information:**

The online version contains supplementary material available at 10.1007/s12275-023-00040-w.

## Introduction

Basal stem rot (BSR) in oil palm (*Elaeis guineensis* Jacq.) is a disease caused by *Ganoderma boninense*, which are bracket fungi belonging to the family Ganodermataceae of the phylum Basidiomycota (Bharudin et al., 2022). As major producers and exporters of palm oil, the Malaysian and Indonesian oil palm industries have been greatly devastated by this BSR disease (Shokrollahi et al., 2021; Susanto et al., 2005), which has also negatively impacted the planting of oil palms in Africa, Cameroon, Colombia, Ghana, Papua New Guinea, Southern Thailand, and Tanzania (Rebitanim et al., 2020). In Malaysia, this disease resulted in an annual loss of up to USD 500 million (Azmi et al., 2020; Ommelna et al., 2012). Climate change is predicted to reduce oil palm productivity beyond 2050 (Paterson et al., 2015, 2017). Temperature increases of 1 to 4 °C may reduce oil palm productivity by 10–41% and make oil palm plantations more susceptible to diseases and pests (Faizah et al., 2022; Sarkar et al., 2020). In addition, the BSR of oil palm is projected to increase, making the industry in Southeast Asia less sustainable (Paterson, 2019a, 2019b, 2020a, 2020b). This disease can seriously compromise world food security. Since palm oil is an essential and versatile raw resource for both the food and non-food industries, the global demand will continue to rise.

Aggressiveness is defined as a quantitative variation in pathogenicity induced by a pathogen strain on its host (Zhan & McDonald, 2013). It was commonly used to assess the quantitative interactions between hosts and pathogens, which includes several components such as infection efficiency, latent period, lesion size, lesion growth rate, sporulation capacity, and pycnidia density (Caffier et al., 2010; Montarry et al., 2008; Zhan et al., 2016). Previous studies have found substantial variation in the aggressiveness levels of *G. boninense* isolated from different regions (Breton et al., 2006; Goh et al., 2014; Idris et al., 2000; Rakib et al., 2015). For example, in Indonesia, Breton et al. (2006) found that the *G. boninense* isolates sampled from several estates varied in their degree of aggressiveness. In Malaysia, the spread rate of *Ganoderma* disease can vary from one estate to another, which may imply differences in *G. boninense* aggressiveness among isolates from different regions (Goh et al., 2014). This was evident when *G. boninense* isolates from different regions in West Malaysia were tested on the vegetative growth of 2- and 5-month-old oil palm seedlings, with 2-month-old seedlings being more responsive in the disease severity index (DSI) and vegetative growth measurement (VGM). Hence, 2-month-old seedlings are preferred for any aggressiveness study (Goh et al., 2014). In Sarawak, Rakib et al. (2015) also found that *Ganoderma* species (*G. boninense* and *G. zonatum*) isolated from Betong and Miri were different in terms of aggressiveness. Therefore, more studies on the aggressiveness of *G. boninense* isolates originated from different regions are crucial for the development of disease management strategies.

According to Breton et al. (2006), discrimination of isolates was done according to their aggressiveness, expressed by the quantification of external and internal disease symptoms using a standardised scoring scale. In the study of the aggressiveness of *G. boninense* isolates, the oil palm seedlings were usually rated for DSI based on a standardized disease class, which is comprised of tissue and morphological symptoms of BSR (Breton et al., 2006; Chan et al., 2011; Goh et al., 2014; Rakib et al., 2015; Shokrollahi et al., 2021). However, Goh et al. (2014) discovered that VGM of infected seedlings was negatively correlated with DSI, implying that VGM could also be used as an additional criterion to assess *G. boninense* aggressiveness. Additionally, re-isolation of *G. boninense* on *Ganoderma* selective medium (GSM) was the most common method used for disease confirmation in infected oil palm seedlings (Breton et al., 2006; Chan et al., 2011; Goh et al., 2014; Idris et al., 2000; Kok et al., 2013; Rakib et al., 2015). However, this method was less accurate as a diagnostic tool for *Ganoderma* detection in infected oil palm because other basidiomycete fungi could also grow on it (Alexander et al., 2014). As a result, supplementary methods such as molecular identification upon re-isolation on GSM should be incorporated to accurately identify the fungal species that causes the disease by sequencing (Bhunjun et al., 2021). Alternatively, the disease confirmation could also be accomplished via the sequencing of polymerase chain reaction (PCR)-amplified fungal DNA extracted from infected seedling tissues, which would be faster than re-isolation. In addition, scanning electron microscopy (SEM) could be used to observe the *G. boninense* hyphae, plant cell alterations (Alexander et al., 2017), or the clamp connection structure (i.e., unique hyphal structures grow in the dikaryotic hyphae during septa development), a typical feature of basidiomycetes in infected tissue of oil palm seedlings, during destructive sampling to confirm the fungal invasion.

To date, culture-based methods, microscopy, and molecular approaches have not been employed in combination to confirm disease presence in *Ganoderma*-inoculated oil palm seedlings. Besides that, the VGM of seedlings inoculated with the isolates could also be an indicator of the aggressiveness of *G. boninense* isolates. Therefore, this study aimed (1) to assess the aggressiveness levels of *G. boninense* isolates collected from BSR diseased oil palms using VGM and DSI of infected oil palm seedlings and (2) to demonstrate the importance of confirming the disease’s presence in infected oil palm seedlings using culture-based methods coupled with microscopy and molecular approaches.

## Materials and Methods

### Strain Collection and Culture Condition

*Ganoderma boninense* isolates used in this study were obtained from infected oil palms at three different estates in Sarawak (Table S1). Isolation was performed according to the method described by Ariffin et al. (2000) and Ariffin and Idris (1991). These isolates were sequenced and identified as *G. boninense* (Midot et al., 2019). The pure cultures of *G. boninense* were maintained as stock cultures on malt extract agar (MEA, Difco) in petri dishes at 16 °C and in sterile tap water at room temperature (27 ± 1 °C) in the dark. Prior to inoculum preparation, *G. boninense* isolates were grown on MEA in petri dishes and incubated at 27 ± 1 °C in the dark for 9 days.

### Inoculum Preparation

Rubberwood blocks (RWBs) of 6 × 6 × 6 cm (Bivi et al., 2016), with a 1 cm diameter hole drilled in the center (Kok et al., 2013), were used to prepare *G. boninense* inocula (Fig. S1A). The RWBs were cleaned and soaked overnight in ultra-pure water (UPW), then autoclaved three times at 121 °C for 45 min. Each RWB was packed in a 7″ × 10″ polypropylene (PP) plastic bag, and 50 ml of malt extract broth (MEB) was added prior to autoclaving at 121 °C for 20 min. A whole petri dish plate of a 9-day-old pure culture of each *G. boninense* isolate on MEA was cut into mycelial plugs and inoculated onto the treated RWBs for incubation in the dark at room temperature (ca. 27 ± 1 °C) for 60 days (Kok et al., 2013).

### Artificial Inoculation of Oil Palm Seedlings

The planting material used in this study was Dura × Pisifera AA Hybrida IS (Applied Agricultural Resources Sdn. Bhd.) (Fig. S1B). The germinated seeds were sown in a black plastic seed tray and maintained in a net house with 50% shade at Kota Samarahan (N1° 28′ 51.55′′, E110° 25′ 28.69′′), Sarawak, Malaysia. The relative humidity and temperature in the net house in which the seedlings were grown ranged from 60 to 76% and 27 to 34 °C, respectively. After 2 months, seedlings were transplanted into 10″ × 12″ black polybags and watered twice daily. Seedlings with uniform growth were chosen for artificial inoculation (Goh et al., 2014), and vegetative growth measurements (VGM) of the 2-month-old seedlings were recorded prior to artificial inoculation. The RWBs that were pre-inoculated with *G. boninense* isolates were placed in a polybag half-filled with planting medium containing a mixture of topsoil, peat, and sand (3:2:1) (Sapak et al., 2008). The roots of the oil palm seedlings were placed in contact with the RWBs that were pre-inoculated with *G. boninense* before planting medium was added to cover the roots (Goh et al., 2014) (Fig. S1C). Each *G. boninense* isolate in this study was tested with three seedlings per replication in five blocks for a total of 15 seedlings per isolate. Seedlings without RWB served as a negative control. A total of 90 seedlings (including the control) were arranged in a randomised complete block design (RCBD) at the net house and fertilized as described by Heriansyah and Tan (2005) for 10 months.

### Aggressiveness Assessment

The vegetative growth measurements, which included the (1) number of leaves, (2) height (cm), (3) bole size (cm), and (4) leaf area (cm^2^), were recorded monthly. The height was measured from the base of the stem (marked with a permanent marker) to the longest leaf tip, and the bole size was measured 1 cm above the soil level with a vernier caliper. Leaf area was estimated and calculated using the following formula: LA = b*(nlw), where b was 0.57, the correction factor; n was the number of leaflets; and lw was the mean of length × mid-width (cm) of the largest leaflet (Corley et al., 1971). The roots were separated, washed, and blotted dry during destructive sampling for fresh root mass (FRM) (g) measurement. Destructive samplings were carried out at 2, 3, 6, 7, and 10 months after inoculation.

The appearance of signs (fungal mycelium and fruiting bodies) and symptoms (necrotic or chlorotic leaves) were recorded weekly. During destructive samplings, the boles of oil palm seedlings were dissected longitudinally and assessed for internal symptoms by a visual estimation of the proportion of tissues damaged by *G. boninense* (Breton et al., 2006). The number of necrotic primary roots was also determined. All the signs and symptoms recorded were used for disease rating. The seedlings were rated based on disease class value (scale of 0–5) adapted from Abdullah et al. (2003) and Nur Sabrina et al. (2012) with a slight modification whereby an additional disease class, class 2, was added to address a disease severity of less than 10% (Table S2).

Disease incidence (DI) is the percentage of diseased plants in the sample, regardless of their severity (Kranz, 1988). Disease development was assessed based on the percentage of disease incidence, using the formula of Campbell and Madden (1990):

$${\text{DI}} = \frac{{{\text{Number}} \;{\text{of}}\;{\text{seedlings}} \;{\text{identified}}\;{\text{as}}\;{\text{diseased}}}}{{{\text{Number}}\;{\text{of}}\;{\text{seedlings}}\;{\text{per}}\;{\text{treatment}}\;{\text{set}}}} \times 100.$$

Disease severity index (DSI) is the percentage of the host tissue or organ covered by symptoms of the diseases (Kranz, 1988). For DSI calculation, the formula of Liu et al. (1995) was used:

$${\text{DSI }} = \frac{{{\text{Number}}\;{\text{of}}\;{\text{plants}}\;{\text{ in}}\;{\text{ the}}\;{\text{ rating}}\; \times \;{\text{ rating}}\;{\text{ number}}}}{{{\text{Total}}\;{\text{ number}}\;{\text{ of}}\;{\text{ plants}} \times {\text{ highest}}\;{\text{ rating}}}} \times 100.$$

The isolates aggressiveness was categorized as in Goh et al. (2014) with slight modifications as follows: (a) highly aggressive (≥ 81%); (b) moderately aggressive (41–80%); (c) less aggressive (21–40%); and (d) least aggressive (0–20%).

### Re-isolation of *G. boninense* on *Ganoderma* selective medium

During the destructive sampling, the seedlings were uprooted and washed carefully under running tap water to remove soil particles. The uprooted seedlings were then split into the bole (cut approximately 5–6 cm from the base of the bole) and the roots. Surface sterilization was carried out in a beaker using 10% commercial bleach containing 5% sodium hypochlorite (Chlorox^®^) for 10 min, followed by 70% ethanol for 5 min. The bole and roots of the seedlings were rinsed with consecutive changes of ultra-pure water. Prior to plating on GSM, the outer bark of the necrotic primary roots and bole of seedlings were immersed in absolute ethanol (HmbG), flamed rapidly, and peeled off. Lastly, the necrotic primary roots and bole were cut into small pieces for plating.

### Microscopic Observation

Sample preparation for scanning electron microscopy (SEM) was performed using the method described by Murtey and Ramasamy (2016) with slight modifications (Fig. S2). The bole and root tissues of the seedlings artificially inoculated with *G. boninense* after 3 months of inoculation were viewed using SEM. Mycelia from reference *G. boninense* pure culture (5A, GbHap1; Accession No. OQ435788) was also prepared for SEM viewing in order to compare the fungal morphology with that of the bole and root tissues.

### Genomic DNA Isolation

DNA extraction of the bole and root tissues and fungal pure cultures isolated from the infected bole and root tissues on GSM were performed using a modified cetyltrimethylammonium bromide (CTAB) method (Voigt et al., 1999). The samples were ground into fine powder in liquid nitrogen using a pestle and mortar, and then resuspended in a 50 ml Falcon tube containing 15 ml of modified CTAB extraction buffer (2% CTAB, 100 mM Tris–HCl, pH 8, 1.4 M NaCl, 20 mM EDTA pH 8, 0.8 mg/ml PVP [polyvinylpyrrolidone], and 1 mg/ml DTT [dithiothreitol]) followed by incubation at 65 °C for 30 min in a shaking water bath BS-31 (Lab Companion). A three-quarters volume of chloroform was added to the CTAB extraction buffer containing the ground materials and inverted for 15 min using an overhead shaker IKA^®^ Trayster basic (IKA-Werke GmbH & Co. KG) and then subsequently centrifuged on a 5430R centrifuge (Eppendorf) at 7000×*g* for 10 min. The aqueous layers were then transferred to a new 50 ml Falcon tube. Ice-cold 99.5% ethanol (two volumes) was added to the aqueous layers and incubated overnight at 4 °C. Any visible DNA strands were transferred into 2 ml microtubes. Excess liquid was removed prior to resuspension of the DNA in 500 μl TE buffer, followed by the addition of 2 μl of 10 mg/ml RNase A. The suspension was incubated for 1 h at 65 °C on a Thermomixer Comfort (Eppendorf). The quality and quantity of the extracted DNA were measured using a NanoPhotometer^®^ P360 (Implen GmbH) and checked by electrophoresis on a 1% agarose gel at 90 V for 35 min. The gel was stained with SYBR^®^ Safe (Invitrogen) and viewed on an Amersham™ Typhoon™ Fluorescent Image Analyzer (GE Healthcare Bio-Sciences AB).

### Molecular Characterization

The DNA extracted from the bole and root tissues were pre-screened using a set of virulent *Ganoderma* specific primers, GanET (5ʹ-GAGTTGTCCCAATAAC-3ʹ) and ITS3 (5ʹ-GCATCGATGAAGAACGCAGC-3ʹ), which were derived from the ITS region of *Ganoderma* with an expected PCR product of 320 bp, similar to *G. boninense* (Bridge et al., 2000). For species identification, extracted DNA was amplified with the universal primers ITS1 (5ʹ-TCCGTAGGTGAACCTGCGG-3ʹ) and ITS4 (5ʹ-TCCTCCGCTTATTGATATGC-3ʹ), approximately 650 bp, adapted from White et al. (1990). Polymerase chain reactions for both primers were performed in a volume of 30 μl containing template DNA (3.0 μl), nuclease free water, colourless Go*Taq*^®^ Flexi buffer (5×) (Promega), MgCl_2_ (25 mM) (Promega), dNTP mix (2.5 mM) (Vivantis Technologies), bovine serum albumin (BSA) (10 mg/ml) (Sigma-Aldrich), forward and reverse primer (10 μM each), and Go*Taq*^®^ DNA polymerase (0.15 μl) (Promega). The PCR protocol for both sets of primers was set as follows: initial denaturation at 95 °C for 2 min; 35 cycles of denaturation at 95 °C for 1 min; annealing at 51 °C for 30 s; extension at 72 °C for 30 s; and a final extension at 72 °C for 5 min. All PCR products were sent for sequencing by commercial sequencing service provider (Next Gene Scientific Sdn. Bhd.). Identification was based on Basic Local Alignment Search Tool (BLAST) searches of ITS sequences against those of deposited ITS sequences in GenBank (National Center for Biotechnology Information, NCBI).

### Statistical Analysis

All statistical analyses were performed using the software RStudio (Version 1.4.1106) (R Core Team, 2021). The Shapiro–Wilk test was used to test the data normality. For normally distributed data, analysis of variance (ANOVA) was used to determine significant differences, followed by Tukey’s test at the 0.05 significance level, while for non-normally distributed data, the Kruskal–Wallis test was utilized, followed by multiple comparisons at 0.05 significance level with the function Kruskal in the package agricolae (Mendiburu & Yaseen, 2020).

## Results

### Vegetative Growth Measurement

**Fig. 1:**
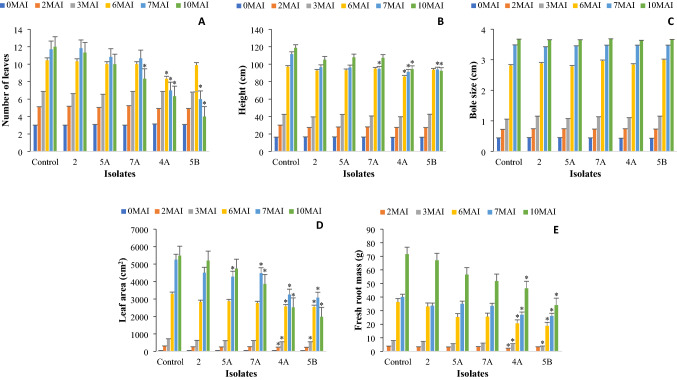
Vegetative growth measurements (VGM) of oil palm seedlings treated with five different *Ganoderma boninense* isolates and the control (without rubber wood block) at 0, 2, 3, 6, 7, and 10 month-after-inoculation (MAI). **A** Number of leaves. **B** Height. **C** Bole size. **D** Leaf area. **E** Fresh root mass. Fresh root mass was quantified during destructive sampling at 2, 3, 6, 7, and 10 month-after-inoculation. The asterisk indicates significant difference from the control at 0.05 significance level

After 2 months of inoculation, there was a reduction in leaf area and fresh root mass of the seedlings inoculated with isolate 4A compared to isolates 5A, 7A, 5B, 2, and uninoculated seedlings (*p* < 0.05) (Fig. 1). However, the vegetative growth of the seedlings inoculated with *G. boninense*, including the number of leaves, height, and bole size, was not significantly different between treatments after 3 months of inoculation (*p* > 0.05). At month six, the seedlings inoculated with 4A had significantly lower (*p* < 0.05) numbers of leaves, height, leaf area, and fresh root mass compared to the uninoculated seedlings. At 7 and 10 months after post-inoculation, the seedlings inoculated with isolates 4A and 5B had significantly lower (*p* < 0.05) numbers of leaves, height, leaf area, and fresh root mass compared to those of the uninoculated seedlings. The bole size showed an increasing trend over time from seedling growth, and none of the bole sizes in the treatments were significantly different from those of the control seedlings throughout the study (*p* > 0.05).

### Disease Signs and Symptoms

**Fig. 2:**
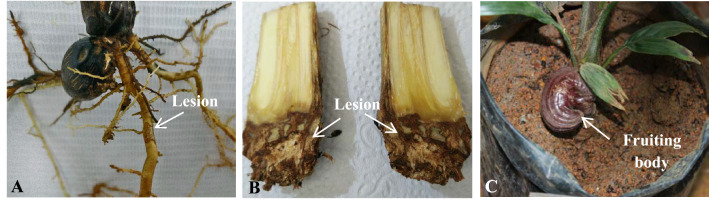
Disease signs and symptoms observed in *Ganoderma boninense* inoculated seedlings. Lesion on the **A** Root **B** Bole, and **C** Fruiting body near the bole of oil palm seedling

During the first 3 months after inoculation, a fungal white mass that eventually formed fruiting bodies was observed near the bole of oil palm seedlings without any leaf symptoms. Later, stunted leaf growth, chlorosis, or necrosis occurred with the presence of fruiting bodies. The distance between the fruiting body and the bole was approximately 0.5–2.0 cm. All five isolates assessed produced lesions on the bole and roots, including fruiting bodies near the bole of the oil palm seedlings (Fig. 2). Isolate 5A was the first isolate that showed symptoms such as the button-like structure near the bole. At the end of the study, only isolate 5B caused death in the seedlings (two out of three seedlings).

### Disease Incidence

**Fig. 3:**
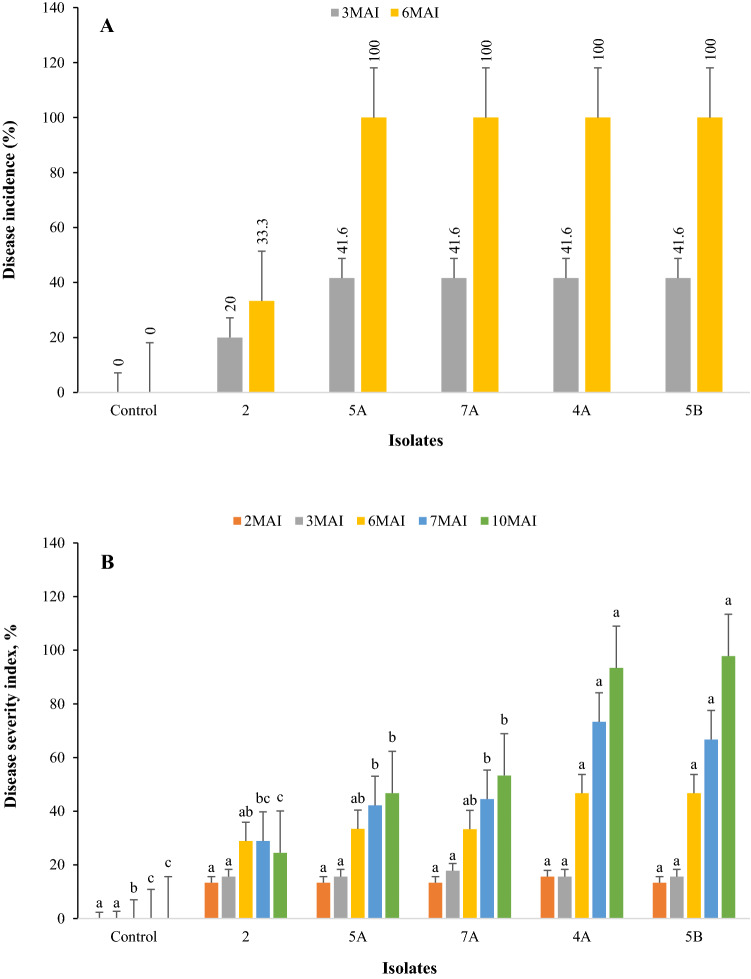
Disease incidence (DI) and disease severity index (DSI) of oil palm seedlings at 2, 3, 6, 7 and 10 month-after-inoculation (MAI) in five different treatments of *Ganoderma boninense*. The same alphabet of the respective month was not significantly different at 0.05 significance level. The error bar represents the standard error

Six months after inoculation, all isolates produced 100% disease incidence except for isolate 2, which produced 100% DI at month seven onward (Fig. 3A).

### Disease Severity Index

Disease severity index (DSI) values for the seedlings inoculated with isolates 5B and 4A were significantly higher compared to those inoculated with the other isolates after 10 months of inoculation (Fig. 3B). After 10 months of inoculation, the death of the seedlings inoculated with 5B and the DSI of 97.8% placed the isolate in the group of highly aggressive according to the classification by Goh et al. (2014). The isolate 4A caused 93.4% disease severity but did not cause the death of the seedlings, thus was grouped as moderately aggressive. Other three isolates, namely 5A, 7A, and 2 were in the group of less aggressive.

### Re-isolation of *G. boninense *on *Ganoderma* Selective Medium

Fungi isolated from infected bole and roots of oil palm seedlings on the GSM plates were identified as either *G. boninense* or non-*Ganoderma* isolates (Table S3 and Fig. S3). Twenty-seven isolates of *G. boninense* were isolated from the infected bole and roots of seedlings challenged with isolates 5A, 7A, 5B, and 4A. Re-isolation of *G. boninense* from the infected bole and roots of seedlings challenged with isolate 2 failed due to slow growth. The GSM plate was overgrown by other fast-growing fungi. Other non-*Ganoderma* species (34 isolates) isolated from GSM were also identified (Fig. S3).

### Microscopic Observation

**Fig. 4:**
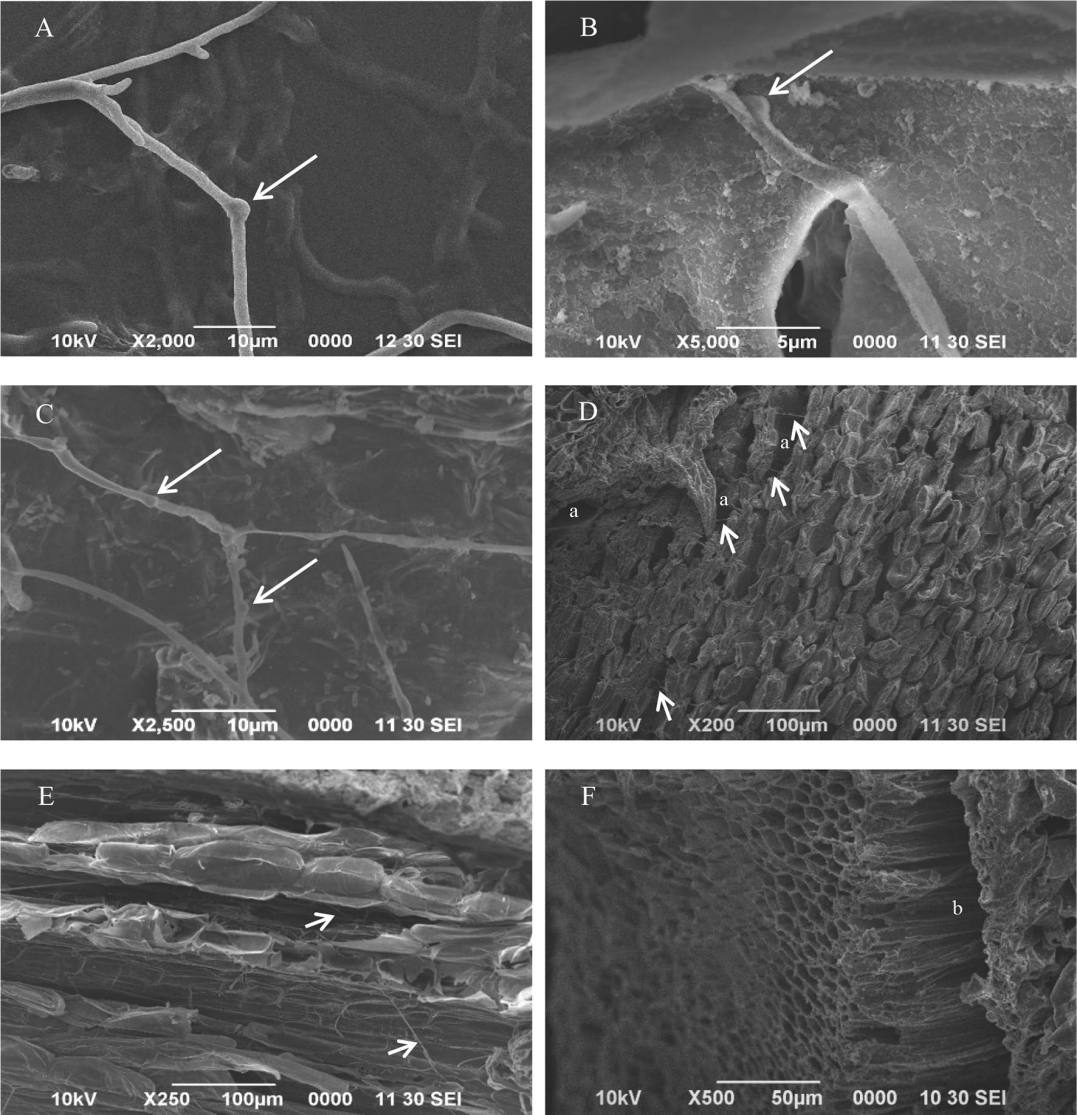
Scanning electron microscopy (SEM) images of *Ganoderma boninense* pure culture hyphal cells, bole, and root tissue of oil palm seedling infected with *G. boninense* isolate 5A (GbHap1, Accession No. OQ435788) after 3 months inoculation. **A** The clamp connection structure (indicated by arrows) of hyphal cells of *G. boninense* pure culture (5A). **B** Infected bole tissues. **C** Infected root tissues. **D** Fungal hyphae (indicated by arrows) and alteration of cell structure (hollow structure indicated as “a”) of the bole. **E** Fungal hyphae in the seedling root (indicated by arrows). **F** Alteration of cell structure (hollow structure indicated as “b”) of the root

Under a scanning electron microscope (SEM), the clamp connection structure that is unique to the fungi in the phylum Basidiomycota was detected in the bole and root tissues of the seedlings artificially inoculated with *G. boninense* (Fig. 4B,C). Aside from that, fungal hyphae and alteration of the cell structure in seedling tissues were also observed (Fig. 4D–F).

### Molecular Characterization

Amplification of the DNA extracted from the bole and root tissues of the artificially inoculated oil palm seedlings using the ITS3/GanET primer pair produced a 320 bp DNA fragment, which was the same size sequenced as the amplicon of DNA from *G. boninense* pure culture (Fig. S4). The identity of the fungi was confirmed as *G. boninense* via the sequencing of the ITS1/ITS4 primer pair amplified products (Table S3).

## Discussion

In this study, all five isolates assessed were confirmed to be pathogenic to oil palm seedlings as they produced lesions on the bole and roots, including the presence of fruiting bodies near the bole of oil palm seedlings. The disease incidence assessment indicated that the signs and symptoms of disease in the artificially inoculated oil palm seedlings manifested as early as 2 months after inoculation in the root zones, where they were in close contact with the pathogen’s inoculum. These tallied with the typical pattern of the mode of infection of *Ganoderma* spp. described by Rees et al. (2007), who indicated that the infection started at the roots through direct contact with the inoculum. After 10 months of inoculation, the majority of the bole tissue of the seedlings inoculated with *G. boninense* was decayed, followed by necrosis of the foliar, which indicated the infection had become severe. Although bole tissue showed signs of decay internally, the bole size remained unaffected throughout this study. This occurs as a result of *G. boninense*'s ability to enter the host through the root and travel internally, causing rot from within without influencing seedling growth initially. Isolate 5B produced the highest DSI (97.8%) 10 months after inoculation and eventually caused the death of the seedlings.

Variations in the level of aggressiveness within *G. boninense* reported in this study correspond to findings by Kok et al. (2013) and Goh et al. (2014), which also found variations in the degree of aggressiveness for 12 different *G. boninense* isolates from West Malaysia. The present study suggests that the variability of aggressiveness in *G. boninense* should not be overlooked. More information on the aggressiveness of *G. boninense* is needed to address the problem effectively. *Ganoderma boninense* from East Malaysia was claimed to be less aggressive compared to that from West Malaysia due to the lower incidence of BSR in East Malaysia and the fact that the majority of the plantations are still of the first generation of oil palm (Chong et al., 2011). However, the data from this study suggest that the aggressiveness of *G. boninense* (5B) isolated from East Malaysia was notable as it could produce a high DSI (97.8%), which suggests that the pathogen’s aggressiveness depends on other factors, for example, the adaptability of the pathogen to its host and environmental conditions, rather than the age of the plantation. The lower incidence of BSR could be due to a lack of BSR incidence being reported, especially among the oil palm smallholders. A survey conducted among the oil palm smallholders in Sabah and Johor showed that most of the smallholders were from the school level of education and had low knowledge related to BSR (Iqlima et al., 2015). Failure to recognise the disease incidence as a result of lack of exposure and understanding of *Ganoderma* disease among oil palm smallholders has attributed to the lower incidence of BSR.

In this study, *G. boninense* inoculation showed a significantly visible effect on plant growth 6 months after inoculation. In other studies, symptoms are evident in seedlings as early as 2 months after inoculation (Goh et al., 2014; Kok et al., 2013). Hasan and Turner (1998) reported that the symptoms could remain absent for years in *Ganoderma*-infected palms until the infection becomes severe. This is due to the slow fungus growth rate through infected roots, which was reported at 1 cm/month in seedling roots; with this rate, the fungus would take 8 years to reach the trunk of a palm (Ariffin et al., 1995; Corley and Tinker, 2015). Foliar symptoms in infected palms start to appear once the internal bole begins decaying and is unable to store nutrients required for the infected seedlings (Rees et al., 2007). In this study, the vegetative growth parameter that was first affected at 2 months was leaf area and fresh root mass, which may not be clearly visible, and at 6 months, leaf number and height. However, the bole size remained unaffected throughout this study. These findings differ slightly from the findings by Sapak et al. (2008), which reported a significant reduction in terms of stem diameter, root biomass, and height as compared to the non-inoculated seedlings in the control.

The fungi isolated from infected seedlings produced brown pigmentation in this study, a pigmentation typical for *Ganoderma* spp. on GSM. However, some of the fungi, when subcultured on MEA, showed different morphology compared to that of the *G. boninense* pure culture on MEA. Since GSM is a semi-selective medium (Alexander et al., 2014), other fungi could also grow on it. Furthermore, *G. boninense* might be present in the bole and root tissues but were unable to grow on the medium, possibly due to its slow growth and being outcompeted by the fast-growing fungi. The incorporation of molecular identification upon re-isolation in this study has increased the precision of disease confirmation. Besides, information on non-*Ganoderma* species obtained from this study could aid in the development of selective media for *Ganoderma* spp..

Furthermore, using molecular approaches, *G. boninense* invasion was confirmed through the amplification of fungal DNA extracted from the bole tissues of the infected seedlings with the ITS3/GanET primers, which produced a 320 bp PCR product. The positive control (DNA from a confirmed *G. boninense* isolate: 5A, GbHap1; Accession No. OQ435788) also produced the same sized amplicon. The ITS3 primer, a universal primer for fungi, was able to minimize the chance of amplifying DNA from other organisms or from the palm itself, while the specificity of the GanET primer also ensures that only oil-palm-associated *Ganoderma* DNA is amplified (Bridge et al., 2000). Besides, PCR sequencing of fungal DNA extracted from disease tissue using the ITS1/ITS4 primer pair further confirmed the presence of *G. boninense* in the infected tissues. Although the SEM method was unable to distinguish the identity of the fungi present in the seedling tissue, images obtained from the SEM provided evidence of fungal invasion. From this study, molecular identification of the fungal DNA extracted from infected seedling tissues for disease confirmation is preferable to re-isolation of fungi because the process takes shorter time. Nonetheless, depending on the research objectives, re-isolation may be required in some circumstances.

Rubber wood blocks, as a source of inoculum, were able to produce successful infection in oil palm seedlings. However, it is not readily available and hard to get, suggesting that an alternative material should be sought and studied. Future studies should use materials that can reduce the time required to induce infection and symptoms, such as immersion of oil palm seedling roots in *G. boninense* mycelial suspension (Purnamasari et al., 2018), with some optimization of suspension concentration and immersion period. It should also be considered to develop selective media using safer ingredients that allow *Ganoderma* rapid growth for re-isolation. A medium that enhances *Ganoderma* growth will allow it to grow faster than other fungi and facilitate *Ganoderma* pure culture isolation.

In the present study, the rubberwood method used to assess the aggressiveness of *Ganoderma* isolates was able to produce infection in the oil palm seedlings. All five isolates assessed had the potential to cause disease in oil palm seedlings; among them, 5B was identified as the most aggressive isolate. It was the only one that caused the death of the seedling. Among the five vegetative growth parameters measured in this study, leaf area, number of leaves, fresh root mass, and height were affected except for bole size. The use of both conventional and molecular approaches allows for precise detection. In the long run, *G. boninense* isolate 5B will be a potential reference isolate for our future research towards contributing new knowledge for *Ganoderma* disease management and control strategies.

## Supplementary Information

Below is the link to the electronic supplementary material.

Supplementary file1 (DOCX 2065 KB)

## Data Availability

Sequence data are deposited in GeneBank with accession numbers stated in the text.
