# Supplementary material for: Comparison of Ganoderma boninense Isolate’s Aggressiveness Using Infected Oil Palm Seedlings
Source: J Microbiol. 2023 Apr 25;61(4):449–59. doi: 10.1007/s12275-023-00040-w (PMC10167175; doi:10.1007/s12275-023-00040-w)
Supplement: Supplementary file 1 — Supplementary file1 (DOCX 2065 KB) [file 12275_2023_40_MOESM1_ESM.docx]

**Supplementary Data**

**Comparison of *Ganoderma boninense* isolate’s aggressiveness using infected oil palm seedlings**

Mei Lieng Lo^[[1]](#footnote-1),^^[[2]](#footnote-2)^ , Tu Anh Vu Thanh2, Frazer Midot1, Sharon Yu Ling Lau1^*^, Wei Chee Wong^[[3]](#footnote-3)^ , Hun Jiat Tung3, Mui Sie Jee1, Mei-Yee Chin1 and Lulie Melling1

**Supplementary data Table S1. *Ganoderma boninense* isolates used for artificial inoculation in this study, the sampling locations, GPS coordinates, and the accession number.**

| **Isolates** | **Location/site** | **GPS coordinates** | **Accession Number** |
| --- | --- | --- | --- |
| 4A | Sg. Meris, Balingian | N 02° 57' 53.1", E 112° 30' 30.0" | OQ435790 |
| 5B | Sg. Meris, Balingian | N 02° 57' 51.7", E 112° 30' 28.8" | OQ435791 |
| 5A | Sg. Liuk, Balingian | N 02° 58' 58.2", E 112° 33' 05.7" | OQ435788 |
| 7A | Sg. Liuk, Balingian | N 02° 59' 51.7", E 112° 32" 13.5" | OQ435789 |
| 2 | Lambir, Miri | N 04° 07' 43.9", E 113° 59' 04.8" | OQ435792 |


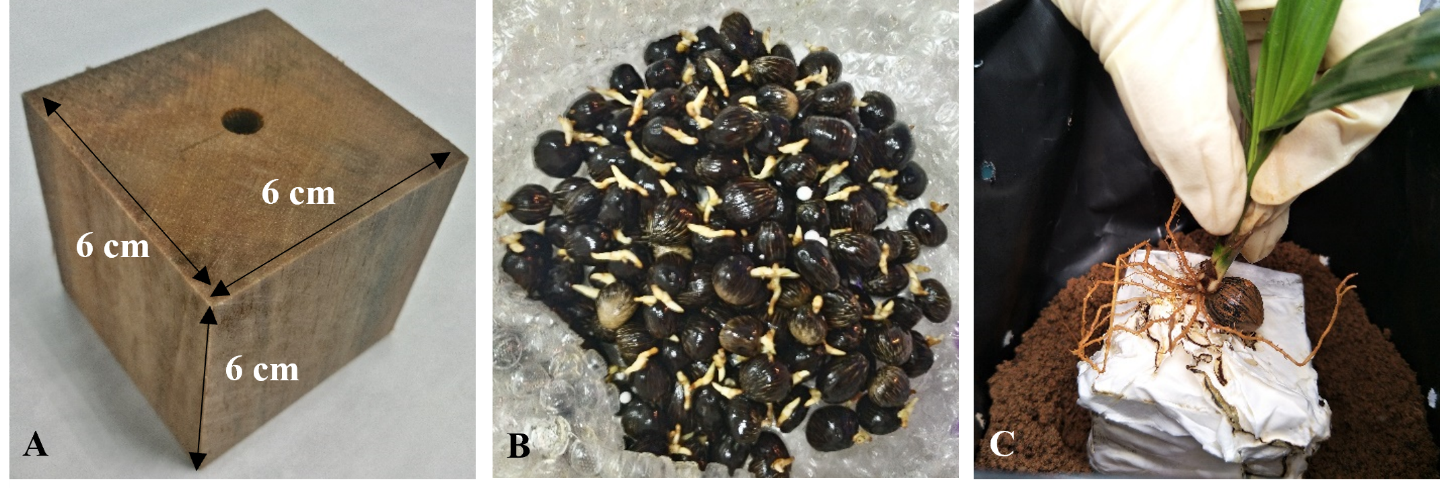


**Supplementary data Fig. S1 (A) The size of rubber wood block (RWB) used in this study. (B) Germinated oil palm seeds (D x P AA Hybrida IS). (C) Artificial inoculation of two months old seedling.**

**Supplementary data Table S2. Disease class value, signs, and symptoms (foliar, bole and root) used for determination of disease severity index.**

| **Disease class value** | **Signs and symptoms on oil palm seedling** |
| --- | --- |
| 0 | Healthy seedlings with green leaves/healthy bole tissue/root tissue without appearance of fungal mycelium on any part of plants |
| 1 | Presence of fungal mycelium or fruiting body on any parts of plants without necrosis or chlorosis leaves/rotting of bole/root tissue |
| 2 | Presence of fungal mycelium or fruiting body on any parts of plants with rotting of necrosis or chlorosis leaves/rotting of bole/root tissue (>1%<10%) |
| 3 | Presence of fungal mycelium or fruiting body on any parts of plants with necrosis or chlorosis leaves/rotting of bole/root tissue (>10%<25%) |
| 4 | Presence of fungal mycelium or fruiting body on any parts of plants with necrosis or chlorosis leaves/rotting of bole/root tissue (>25%<75%) |
| 5 | Presence of fungal mycelium or fruiting body on any parts of plants with necrosis or chlorosis leaves/rotting of bole/root tissue (>75%) or seedling dead |


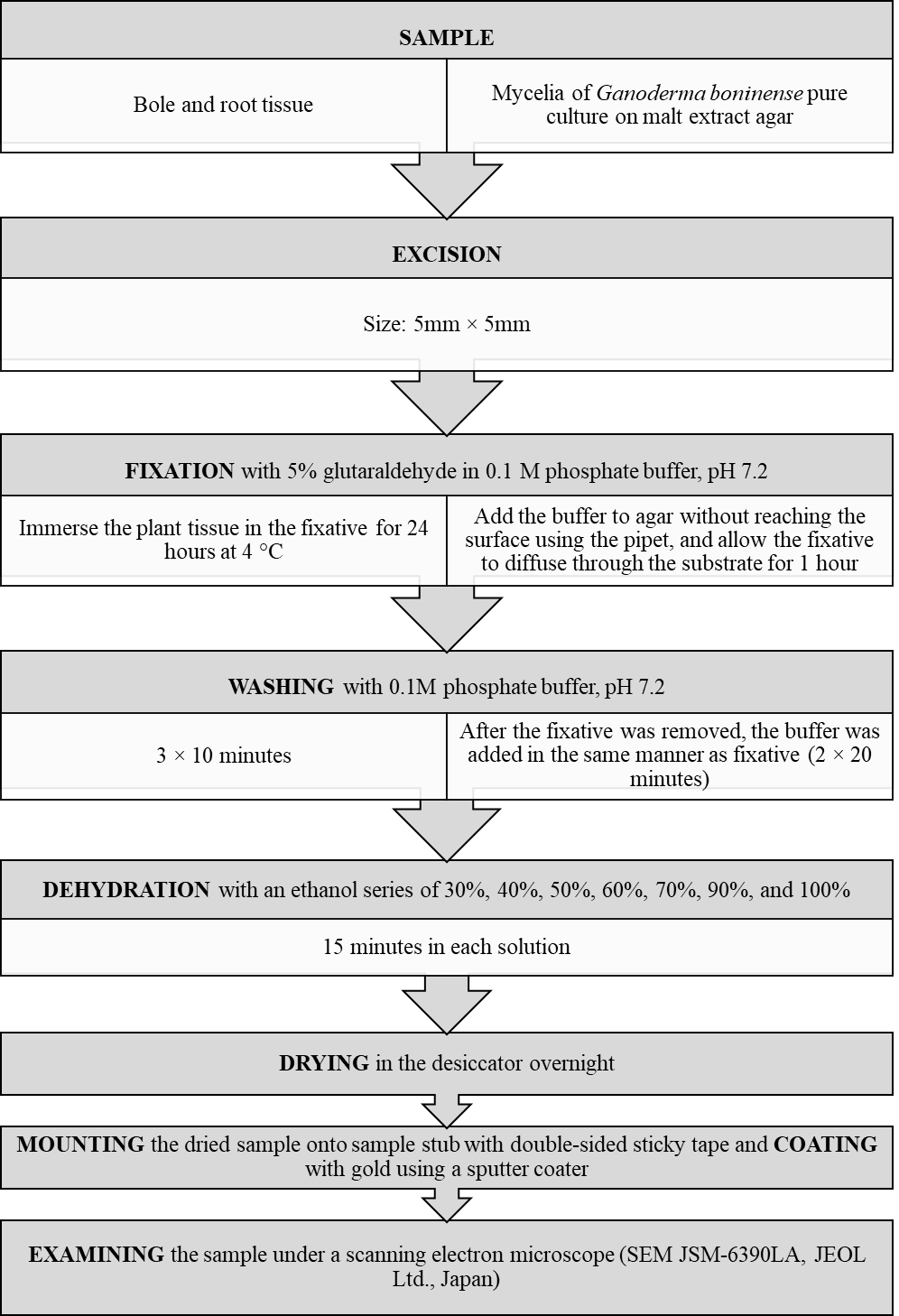


**Supplementary data Fig. S2 Sample preparation for scanning electron microscope (SEM) as described by Murtey and Ramasamy (2016) with slight modifications.**

**Supplementary data Table S3. Sequencing results of ITS1/ITS4 amplified DNA extracted from the root and bole tissue of *Ganoderma boninense* infected seedlings and those re-isolated on *Ganoderma* selective medium (GSM).** Identification was based on BLAST searches of ITS sequences against those of deposited ITS sequences in GenBank (NCBI).

| **Source** | **Isolate** | **Sample ID.** | | **Identity** | **Similarity** | **GenBank Accession no.** |
| --- | --- | --- | --- | --- | --- | --- |
| **Infected Seedlings** | 5A | R7 | Root | *Ganoderma boninense* | 100.00% | OQ435793 |
|  |  | R10 |  | *Ganoderma boninense* | 100.00% | OQ435794 |
|  | 7A | R26 |  | *Ganoderma boninense* | 100.00% | OQ435795 |
|  |  | R30 |  | *Ganoderma boninense* | 100.00% | OQ435796 |
|  |  | R33 |  | *Ganoderma boninense* | 100.00% | OQ435797 |
|  |  | R36 |  | *Ganoderma boninense* | 100.00% | OQ435798 |
|  | 5B | R41 |  | *Ganoderma boninense* | 100.00% | OQ435799 |
|  |  | R51 |  | *Ganoderma boninense* | 100.00% | OQ435800 |
|  |  | R53 |  | *Ganoderma boninense* | 100.00% | OQ435801 |
|  | 4A | R60 |  | *Ganoderma boninense* | 100.00% | OQ435802 |
|  |  | R66 |  | *Ganoderma boninense* | 100.00% | OQ435803 |
|  |  | R70 |  | *Ganoderma boninense* | 100.00% | OQ435804 |
|  |  | R72 |  | *Ganoderma boninense* | 100.00% | OQ435805 |
|  | 5A | B10 | Bole | *Ganoderma boninense* | 100.00% | OQ435806 |
|  |  | B13 |  | *Ganoderma boninense* | 100.00% | OQ435807 |
|  | 7A | B22 |  | *Ganoderma boninense* | 100.00% | OQ435808 |
|  |  | B26 |  | *Ganoderma boninense* | 100.00% | OQ435809 |
|  | 5B | B54 |  | *Ganoderma boninense* | 100.00% | OQ435810 |
|  | 4A | B60 |  | *Ganoderma boninense* | 100.00% | OQ435811 |
|  |  | B64 |  | *Ganoderma boninense* | 100.00% | OQ435812 |
|  |  | B70 |  | *Ganoderma boninense* | 100.00% | OQ435813 |
| ***Ganoderma* selective medium** | 5A | R10 | Root | *Ganoderma boninense* | 100.00% | OQ435814 |
|  |  | R14 |  | *Ganoderma boninense* | 100.00% | OQ435815 |
|  |  | R17 |  | *Ganoderma boninense* | 100.00% | OQ435816 |
|  | 7A | R25 |  | *Ganoderma boninense* | 100.00% | OQ435817 |
|  |  | R26 |  | *Ganoderma boninense* | 100.00% | OQ435818 |
|  | 5B | R40 |  | *Ganoderma boninense* | 100.00% | OQ435819 |
|  |  | R51 |  | *Ganoderma boninense* | 100.00% | OQ435820 |
|  |  | R52 |  | *Ganoderma boninense* | 100.00% | OQ435821 |
|  |  | R54 |  | *Ganoderma boninense* | 100.00% | OQ435822 |
|  | 4A | R61 |  | *Ganoderma boninense* | 100.00% | OQ435823 |
|  |  | R70 |  | *Ganoderma boninense* | 100.00% | OQ435824 |
|  |  | R71 |  | *Ganoderma boninense* | 100.00% | OQ435825 |
|  | 5A | B10 | Bole | *Ganoderma boninense* | 100.00% | OQ435826 |
|  |  | B14 |  | *Ganoderma boninense* | 100.00% | OQ435827 |
|  | 7A | B26 |  | *Ganoderma boninense* | 100.00% | OQ435828 |
|  | 5B | B47 |  | *Ganoderma boninense* | 100.00% | OQ435829 |
|  |  | B51 |  | *Ganoderma boninense* | 100.00% | OQ435830 |
|  |  | B52 |  | *Ganoderma boninense* | 100.00% | OQ435831 |
|  |  | B53 |  | *Ganoderma boninense* | 100.00% | OQ435832 |
|  |  | B54 |  | *Ganoderma boninense* | 100.00% | OQ435833 |
|  | 4A | B60 |  | *Ganoderma boninense* | 100.00% | OQ435834 |
|  |  | B61 |  | *Ganoderma boninense* | 100.00% | OQ435835 |
|  |  | B64 |  | *Ganoderma boninense* | 100.00% | OQ435836 |
|  |  | B68 |  | *Ganoderma boninense* | 100.00% | OQ435837 |
|  |  | B69 |  | *Ganoderma boninense* | 100.00% | OQ435838 |
|  |  | B70 |  | *Ganoderma boninense* | 100.00% | OQ435839 |
|  |  | B71 |  | *Ganoderma boninense* | 100.00% | OQ435840 |

**
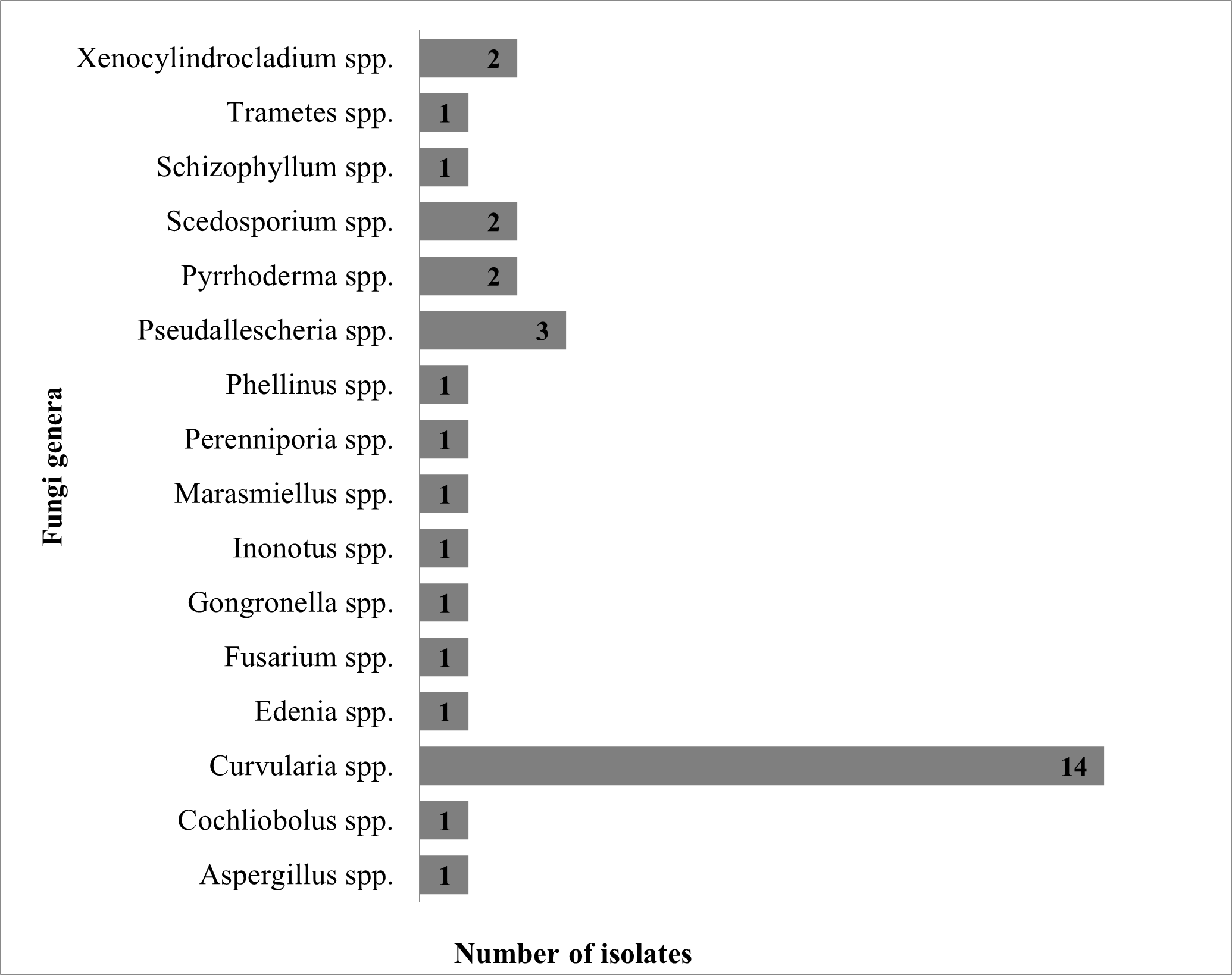
**

**Supplementary data Fig. S3. Fungus isolated from *Ganoderma* selective medium (GSM).**

**
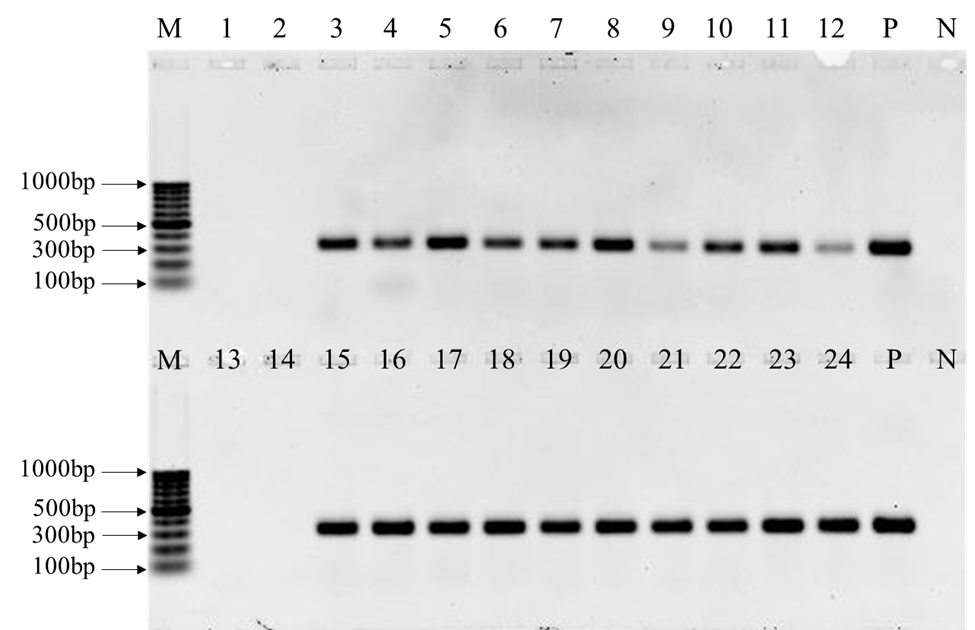
**

**Supplementary data Fig. S4. PCR products of DNA extracted from infected seedlings produced 320 bp DNA fragment on a 2% agarose gel.** Legend: Lane M: Molecular weight ladder (100 bp); lanes 1–12: infected bole tissues; lanes 13–24: infected root tissues; lane P: positive control (DNA from confirmed *G. boninense* isolate: 5A, GbHap1; Accession No. OQ435788) and lane N: negative control (sterile ultra pure water). The 320 bp DNA band was absent for the DNA extracted from infected bole and root tissues of seedlings inoculated with isolates 2 (bole: lane 1–2; root: lane 13–14).

1. Sarawak Tropical Peat Research Institute, Lot 6035, Kuching-Kota Samarahan Expressway, 94300 Kota Samarahan, Sarawak, Malaysia [↑](#footnote-ref-1)
2. Faculty of Resource Science and Technology, Universiti Malaysia Sarawak, Jalan Datuk Mohammad Musa, 94300 Kota Samarahan, Sarawak, Malaysia [↑](#footnote-ref-2)
3. Applied Agricultural Resources Sdn. Bhd., Kota Damansara, 47810, Petaling Jaya, Selangor, Malaysia

   ^*^ For correspondence. E-mail: lauyuling@gmail.com; Tel.: +6082662491; Fax: +6082663615 [↑](#footnote-ref-3)
